# Exploring control-value motivational profiles of mathematics anxiety, self-concept, and interest in adolescents

**DOI:** 10.3389/fpsyg.2023.1140924

**Published:** 2023-04-17

**Authors:** Michael D. Broda, Erica Ross, Nicole Sorhagen, Eric Ekholm

**Affiliations:** ^1^Virginia Commonwealth University, Richmond, VA, United States; ^2^Millersville University, Millersville, PA, United States; ^3^Chesterfield County Public Schools, Chesterfield, VA, United States

**Keywords:** control-value theory, math anxiety, academic emotions and attitudes, self-concept (academic), latent profile analysis

## Abstract

In this study, we identified multidimensional profiles in students’ math anxiety, math self-concept, and math interest using data from a large generalizable sample of 16,547 9th grade students in the United States who participated in the National Study of Learning Mindsets. We also analyzed the extent that students’ profile memberships are associated with related measures such as prior mathematics achievement, academic stress, and challenge-seeking behavior. Five multidimensional profiles were identified: two profiles which demonstrated relatively high levels of interest and self-concept, along with low math anxiety, in line with the tenets of the control-value theory of academic emotions (C-VTAE); two profiles which demonstrated relatively low levels of interest and self-concept, and high levels of math anxiety (again in accordance with C-VTAE); and one profile, comprising more than 37% of the total sample, which demonstrated medium levels of interest, high levels of self-concept, and medium levels of anxiety. All five profiles varied significantly from one another in their association with the distal variables of challenge seeking behavior, prior mathematics achievement, and academic stress. This study contributes to the literature on math anxiety, self-concept, and interest by identifying and validating student profiles that mainly align with the control-value theory of academic emotions in a large, generalizable sample.

## 1. Introduction

For decades, math and other STEM disciplines have been at the forefront of many educational research and reform efforts (National Research Council et al., 2012; National Academies of Sciences, Engineering, and Medicine et al., 2019). In a broad sense, the goals of these efforts are to better prepare students to understand STEM concepts they may encounter in their daily lives and/or to prepare them for careers in STEM disciplines. One commonality among many of these approaches to reforming STEM is an acknowledgment that, though academic achievement is important, it is far from the only factor that contributes to STEM learning and expertise (National Academies of Sciences, Engineering, and Medicine, 2019). Thus, a multi-dimensional perspective on STEM learning is vital to achieving these shared goals.

To extend this multi-dimensional perspective, the current project extends on previous research related to student STEM learning by applying constructs related to the control-value theory of academic emotions (Pekrun, 2006) and employing latent profile analysis (LPA), a person-centered approach capable of capturing within-person relations among several variables. More specifically, we estimate profiles of a nationally representative sample of United States high school students as indicated by their math self-concept, math interest, and math anxiety. Finally, we examine the extent to which student profile membership is associated with prior math achievement, as well as academic stress and challenge-seeking behaviors. This work can inform future mathematics curriculum and intervention design by highlighting commonalities and differences in students’ motivational and emotional profiles, as well as demonstrating how these profiles may be associated with their own prior experiences in math.

In the sections that follow, we first situate this work in Control-Value Theory of Emotions (Pekrun, 2006; Pekrun et al., 2007). We then review relevant literature on students’ motivation and learning as related to theory. Finally, we illustrate the contribution of within-person methods such as LPA to work in similar fields and contexts.

## 2. Review of relevant literature

### 2.1. The control-value theory of achievement emotions

The control-value theory of achievement emotions (C-VTAE) provides a framework explaining how emotions are tied to achievement activities and outcomes. Pekrun (2006) identifies a theoretical co-development of both control and value appraisal. First, control-value theory posits that students’ academic emotions—both state-level and trait-level emotions—arise due to control beliefs and value beliefs. In other words, the specific achievement emotions students will feel when engaged with an academic task depend on the extent to which they feel in control of (or out of control of) a task as well as the extent to which the task is important to them (Pekrun et al., 2007). Further, these control beliefs, value beliefs, and the subsequent emotions they elicit are thought to be domain-specific (Pekrun et al., 2002; Pekrun, 2006; Boekaerts and Pekrun, 2015).

Through this framework, math anxiety is hypothesized to develop when a student experiences low control of a math activity, especially when also experiencing a higher value appraisal (Nie et al., 2011; Bieg et al., 2013; Wang et al., 2021). Hence, students experiencing the most math anxiety would be those who are experiencing low control (in our case, operationalized by self-concept), along with high value (operationalized in this study by math interest). Although several studies related to the components of control and value appraisal have found mixed findings (e.g., Ganley and McGraw, 2016; Malanchini et al., 2020), most studies find that control appraisal (i.e., self-concept) and value appraisal (i.e., math interest) have negative associations with math anxiety (Ahmed et al., 2012; Ganley and McGraw, 2016; Muis et al., 2018; Wang et al., 2021). Many studies have also found that math anxiety is negatively related to math achievement, both concurrently and longitudinally (Ma, 1999; Pekrun et al., 2017; Namkung et al., 2019), with potentially reciprocal links between these constructs. The presence of other negative achievement emotions, such as anger or boredom, may also impact the strength of association between anxiety and math achievement (Peixoto et al., 2017; Abín et al., 2020).

When both control and value appraisal are studied together, the negative association between math self-concept and anxiety is stronger when the student’s math value is perceived as higher (Bieg et al., 2013). The recent study developed by Wang et al. (2021) establishes how math anxiety, self-concept, and math value develop in tandem over time, finding heterogeneous contributions of both control and value appraisal. Wang et al. (2021) found three classes of co-development with varying relationships between anxiety, self-concept and math value. This included a stable class, characterized by stable anxiety, high concept, and high value. They also found a class that had increasing anxiety, along with decreasing self-concept and value. The third class they described as fluctuating with curvilinear changes in math anxiety, self-concept, and value.

Relatedly, Orbach and Fritz (2022) used latent profile analysis to identify different patterns of trait math anxiety and state math anxiety while also analyzing attitudes toward math, academic self-concept, fixed/growth mindsets, executive functions, and math performance. Their results found seven distinct profiles of math anxiety and core beliefs toward math. The profile with lower math performance had higher anxiety and negative cognitive beliefs towards math, and the profile with higher math performance had high trait math anxiety and positive cognitive beliefs. These two recent studies highlight the complex interplay between these constructs as well as the importance of using within-person analyses to better understand how these constructs function differently for different people.

### 2.2. Math anxiety

According to Ramirez and colleagues (Ramirez et al., 2018), math anxiety “refers to feelings of fear, tension, and apprehension that many people experience when engaging with math” (p. 145). This anxiety is theorized to be domain-specific and trait-like, suggesting that these feelings are relatively consistent across mathematics tasks and are constrained to the domain of mathematics (although people may nevertheless experience anxiety in other domains). Consistent with other emotions, math anxiety may have affective, conative, cognitive, physiological, and neurological components (Pekrun, 2006; Lyons and Beilock, 2012; Boekaerts and Pekrun, 2015; Ramirez et al., 2018). For example, when presented with a math assignment, a student with high math anxiety might worry that he will fail on the assignment, he might be motivated to avoid the assignment as a way to regulate this anxiety (Gross, 2015), he might sweat or experience an increased heart rate, and he might experience similar neural activation patterns to a person in physical pain (Lyons and Beilock, 2012).

Numerous studies have found that math anxiety is negatively related to math achievement, both concurrently and longitudinally (Ma, 1999; Pekrun et al., 2017; Namkung et al., 2019), with potentially reciprocal links between these constructs. Several theories exist that explain the relations between math anxiety and math achievement; however, we focus here on Pekrun’s CVT (Pekrun, 2006; Pekrun et al., 2007, 2011), since it can account for other constructs relevant to the current study.

Performing poorly on math tasks can diminish learners’ math self-efficacy beliefs, or their beliefs about their ability to succeed on math tasks (Bandura, 1986; Usher and Pajares, 2008). In other words, past failures in math may lead students to doubt their ability to succeed in math in the future. According to the control-value theory of achievement emotions (Pekrun, 2006), such low control beliefs, particularly when combined with high value beliefs, likely lead learners to experience math anxiety. That is, when learners consider performing well in math to be valuable but also doubt their ability to do so, they are likely to feel anxiety. A recent large-scale longitudinal study of secondary students conducted by Pekrun and colleagues (Pekrun et al., 2017) supports the notion of reciprocal causality between math achievement and math anxiety. Across all time points in a five-year study, math anxiety modestly yet significantly predicted later math achievement; likewise, math achievement modestly yet significantly predicted later math anxiety.

Although much of this research attests that math anxiety may disrupt learning and tends to have an overall negative effect on math performance, there may be some cases in which some math anxiety may be beneficial (Wang et al., 2015). Famously, the Yerkes-Dodson law (Yerkes and Dodson, 1908) suggests that the relationship between psychological arousal and task performance is curvilinear, meaning that, up to some point, increased anxiety may relate to improved performance on math tasks. Zeidner (1998) noted that this effect likely depends upon task difficulty, with performance on more difficult tasks improving less or not at all even with modest increases in anxiety, and performance on simpler tasks showing more benefit from increased anxiety.

### 2.3. Math interest

Interest refers to “the psychological state of engaging or the predisposition to re-engage with particular classes of objects, events, or ideas over time” (Hidi and Renninger, 2006, p. 112). Much like other motivational variables in academic contexts, interest is domain-or content-specific, and, according to Frenzel and colleagues (Frenzel et al., 2010), “there is no such thing as general student interest” (p. 509). Although students can and often do experience state-like interest, often referred to as situational interest, which is interest that is triggered by situation-specific factors (e.g., a student might be interested in a specific math lesson because the teacher uses a novel technology or demonstration), the current study is more concerned with trait-like interest, sometimes referred to as individual interest, which is a more enduring disposition (Ainley, 2007). Nevertheless, it is important to note that triggering situational interest is a critical early step in developing individual interest in specific topics or domains (Hidi and Renninger, 2006; Ainley, 2007). Individual interest also aligns closely with conceptualizations of intrinsic value (Wigfield and Eccles, 2000; Wigfield and Cambria, 2010), which refers to the enjoyment a student gains from engaging with an academic task.

Across content areas, interest in a specific subject is positively related to achievement, engagement with tasks in that subject, attention, goal-setting, competence beliefs, and expertise development (Schiefele et al., 1992; Alexander et al., 1997; Alexander and Murphy, 1998; Schiefele, 1999, 2001; Ainley et al., 2002; Alexander, 2003; Ainley, 2007; Wigfield and Cambria, 2010). However, previous research suggests that over time, students may lose interest in mathematics as they progress through school (Köller et al., 2001; Fredricks and Eccles, 2002; Frenzel et al., 2010). Furthermore, students’ levels of interest seem to be influenced by classroom-level factors, including teachers’ enthusiasm for math (Frenzel et al., 2009, 2010).

Interest may facilitate deeper processing and more sustained engagement with tasks in that area (Murphy and Alexander, 2002; Alexander, 2003). Interest, then, may promote a deeper understanding of math topics, more strategic engagement with tasks, and the choice of enrolling in more challenging math courses (Köller et al., 2001), all of which contribute to the STEM expertise currently being called for by national policymakers (National Research Council et al., 2012; National Academies of Sciences, Engineering, and Medicine et al., 2019).

### 2.4. Students’ math self-concept

Self-concept refers to the perception one has of one’s self through experiences and how they interact and interpret their environment (Shavelson et al., 1976). Self-concept beliefs can measure how one views their own abilities in academic or nonacademic domains (Marsh and Craven, 1996). Assessments of self-concept include examples such as “Mathematics is one of my best subjects” and “It’s important to me to do well in mathematics classes” (SDQI; Marsh et al., 2005). Recent work has identified self-concept as a mediating factor between math performance, anxiety, though it has focused on elementary rather than secondary school settings (Justicia-Galiano et al., 2017; Kaskens et al., 2020). Prior research on students’ math self-concept in secondary settings has identified a positive relationship between self-concept and achievement (Marsh and Martin, 2010; Parker et al., 2013). For example, Timmerman et al. (2017) used correlation and regression analyses to examine the association between math anxiety, test anxiety, math self-concept, achievement motivation, and the outcome of math achievement in a sample of high school students in Netherlands. They found that when considered concurrently, only math self-concept contributed unique explanatory power when predicting math achievement. Trait anxiety, such as math anxiety, has been found to be negatively relative to self-concept (Ahmed et al., 2012).

### 2.5. Person-centered approaches to studying mathematics motivation

Person-centered approaches such as latent profile analysis (LPA; Masyn, 2013; Nylund-Gibson and Choi, 2018) and cluster analysis (Everitt, 1979) attempt to identify relatively homogeneous groups of people within a heterogeneous population or sample by differentiating them according to their scores on several key variables. In doing so, these methods can provide researchers with a more nuanced understanding of within-person relations among math motivational constructs that other approaches, such as examining bivariate correlations or linear predictive relations, may not adequately capture.

For example, in a study investigating Taiwanese junior high school students’ conceptualizations of what comprises learning in math, Wang and colleagues (Wang et al., 2017) used LPA to identify four different student learning profiles, which in turn were associated with differences in both mathematics self-efficacy and performance. Wang et al. (2018) used a similar approach to identify latent profiles of math anxiety (both learning anxiety and exam anxiety) and mathematics motivation. Furthermore, LPA has been used to investigate a wide range of educational phenomena, including profiles of middle-school students’ science motivational beliefs (Bae and DeBusk-Lane, 2018), secondary students’ sources of science self-efficacy (Chen and Usher, 2013), math achievement and anxiety (Hart et al., 2016), math motivation, engagement, and academic performance (Xie et al., 2020), and students’ personal conceptions of math learning (Wang et al., 2017).

This study will also utilize LPA to better understand the relationship between math anxiety, self-concept, and interest in a nationally representative sample of 9th grade students in the United States. This study will also determine the extent to which membership in each profile is associated with academic stress, challenge seeking behaviors, prior mathematics GPA. Given the demonstrated salience of math motivation and the complex interplay of control, value, and emotions demonstrated by C-VTAE, and the importance of measuring it with person-centered approaches, our work sought to answer the following research questions:

### 2.6. Research questions and hypotheses

1. Are there distinct profiles, or classes, of students who show similar patterns of performance across measures of math anxiety, math self-concept, and math interest?

Hypothesis 1: Given prior literature using related constructs and the theoretical relationship between anxiety, math self-concept, and math interest articulated in C-VTAE, we hypothesize that students in this sample can be classified into a number of distinct latent profiles. Given the exploratory focus of this paper, we do not offer a hypothesis as to the number of profiles which may exist.

1. To what extent are students’ profile memberships associated with related measures, including prior mathematics achievement, perceived academic stress and challenge-seeking behavior?

Hypothesis 2: Our hypothesis for this question is more general in nature, given the exploratory purpose of the paper. We would hypothesize, based on the theoretical relationships between our variables of interest (math interest, anxiety, and self-concept) and prior math achievement, academic stress, and challenge-seeking behavior, that latent profiles defined by our variables of interest would also exhibit significant differences on these related constructs.

## 3. Methods

### 3.1. Data

Data for this paper come from a nationally generalizable dataset collected as part of the National Study of Learning Mindsets (Yeager et al., 2019). These data included responses from 16,547 students nested within 76 regular public high schools in the United States. Schools were selected for participation based on a two-stage probability sampling procedure outlined in Tipton et al. (2019). One hundred and thirty nine high schools from across the United States were selected for recruitment into the study; 76 agreed and eventually participated in the NSLM. Gopalan and Tipton (2018) conducted numerous statistical comparisons of the schools that participated in the NSLM with national benchmarks and found that the NSLM sample closely resembles the profile of “all regular, U.S. public high schools with at least 25 students in 9th grade and in which 9th grade is the lowest grade” (p. 1).

Data used in the current study were all collected online via a restricted web survey portal in Fall 2015. Students were asked to complete a 25 min survey using computers provided by their school. Table 1 provides school-and student-level descriptive statistics and demographic characteristics of the sample. The data are now publicly available (Yeager, 2015) *via* application to the Inter-university Consortium for Political and Social Research (ICPSR).

**Table 1:** Student and school-level descriptive statistics for analytic sample.

Variable	Mean/%	SD	Min	Max
School-level				
School average math PSAT Pct	47.61	6.08	29	58
NAEP math 2013 (Std. scores)	0.04	0.71	−1.56	1.83
Prop. AP test takers	0.01	0.02	0.00	0.08
Prop. Free/reduced lunch	0.37	0.23	00.00	0.94
Prop. White	0.66	0.28	0.01	1.00
Prop. below federal poverty line	0.19	0.09	0.04	0.44
Total enrollment	1011.27	716.68	120.00	2807.00
Student-level				
Female	49.80%	-	-	-
Male	50.20%	-	-	-
Asian	4.80%	-	-	-
Black	15.60%	-	-	-
Hispanic	18.80%	-	-	-
Native American	0.80%	-	-	-
Multi-race	2.90%	-	-	-
Pacific islander/native Hawaiian	2.10%	-	-	-
White	55.10%			
Qualify for FRL	36.60%			
Math interest	2.68	1.15	1.00	5.00
Math anxiety	2.51	1.14	1.00	5.00
Prior math GPA	2.91	0.95	0.23	4.30
Math self concept	5.22	1.18	1.00	7.00
Academic stress	3.08	1.18	1.00	5.00
Challenge-seeking behavior (1 = Yes, 0 = No)	43.50%	-	-	-

*N* (schools) = 76. *N* (students) = 16,547. PSAT, preliminary scholastic achievement test. NAEP, national assessment of educational progress. GPA, grade point average. FRL, free and reduced lunch.

### 3.2. Measures

Student measures used in this study included: (1) prior math achievement, (2) math anxiety, (3) math interest, (4) math self-concept, (5) math challenge-seeking behavior, and (6) perceived academic stress. Math prior achievement was operationalized as each students’ 8th grade math grade point average (GPA). This measure ranged from 0 (F) to 4.30 (A).

Math anxiety (“Math makes me anxious”), interest (“Math is interesting to me”), and academic stress (“Schoolwork is stressful for me”) were all measured single items from the time-1 student survey, with a 5-point Likert-type response scale that ranged from 1 (“Strongly disagree”) to 5 (“Strongly agree”). Math self-concept (“I am a math person”) was a single item ranging from 1 (“Strongly disagree”) to 7 (“Strongly agree”). Mathematics challenge seeking behavior was also measured using a single item, which described a challenging math problem and then asked the student how likely they were to try and solve the problem. Responses here were also measured on a 0 (“Highly unlikely”) to 4 (“Highly likely”) Likert-type scale. Given that items used somewhat different scale lengths, for purposes of interpreting profile differences we used a percent of maximum possible (Cohen et al., 1999) transformation, which converts differing scale lengths into a percent of total possible endorsement of each item.

### 3.3. Statistical analyses

Latent profile analysis is a method that attempts to identify hidden subgroups within a sample or population (Nylund-Gibson and Choi, 2018) and is analogous to factor analysis approaches. Where factor analysis attempts to use participant response patterns on specific items to identify one or more latent factors measured by these items, LPA uses participant response patterns on items to identify one or more (typically more) hidden groups of people. This emphasis on identifying subgroups of people has led many to refer to LPA (and LCA) as a “person-centered approach” to analysis, where factor analysis may be considered a “variable-centered approach” (Nylund and Asparouhov, 2007; Berlin et al., 2014). As such, LPA may offer insights into typologies of people that traditional variable-centered approaches may fail to identify.

The main variables of interest used in our LPA were mathematics self-concept, mathematics anxiety, and mathematics interest, all measured by self-report items and described above. These three indicators are the basis for determining the optimal number of student subgroups to answer Research Question (RQ) 1. As described in RQ 2, several additional measures were used as distal outcomes to test the validity of the LPA solution, including academic stress and challenge-seeking behavior, both measured by self-report items, and prior math achievement, as measured by students’ 8th grade mathematics grade point average (GPA).

The LPA modeling approach used was the parametric procedure outlined by Finch and French (2014). To answer RQ 1, single-level LPA was used to determine the appropriate number of student profiles. The fit statistics used to make this determination included: the Bayesian Information Criterion (Schwarz, 1978), the sample size adjusted Bayesian Information Criterion (Sclove, 1987), the Bootstrap Likelihood Ratio Test (McLachlan et al., 2019), the Lo–Mendell–Rubin Test (Lo et al., 2001), consistent AIC (Bozdogan, 1987), Bayes factor (Wagenmakers, 2007), approximate weight of evidence (Banfield and Raftery, 1993) and approximate correct model probability (Schwarz, 1978). To answer RQ2, the distal outcomes described above were included following the Bolck, Croons, and Hagenaars (BCH; Bolck et al., 2004) process for modeling outcomes in an LPA recommended by Nylund-Gibson et al. (2019). The BCH method adjusts associations between profile membership and distal outcomes for potential classification error. In this approach the parameters of the latent classes are held fixed while also accounting for classification error. Then, distal outcomes are included and their relation to the latent class variable is estimated by comparing class-specific mean and variance estimates among the classes. This is accomplished *via* a global Chi-square test, which is significant if any pairwise comparisons are significant, as well as *via* individual pairwise comparisons.

Several additional methodological challenges were addressed using the following approaches. To account for possible violations of the multivariate normality assumption, a robust maximum likelihood estimator (Estimator = *MLR* in *Mplus*) was used. To assess model fit, the scaled Satorra-Bentler Chi-Square statistic (Satorra and Bentler, 2001) was used in place of the traditional Chi-Square, which is not robust to violations of normality. Standard errors were adjusted for the clustering of students within schools using the sandwich estimator (Huber, 1967; White, 1980).

## 4. Results

Results are presented in two sections: (a) enumerating the mathematics learning profile groups (latent profiles) and (b) assessing mathematics learning profile differences with respect to distal measures.

### 4.1. Identifying learning profiles

Table 2 provides model fit statistics for 1-to-10 profile solutions. Overall, fit statistics suggested a five-profile solution provided the best model fit, as indicated by the nonsignificant VLMR-LRT test results for the 6-profile and greater solutions. The BIC, and SABIC, CAIC, and AWE statistics all dropped consistently from 1 to 10, offering no additional evidence. This is common when working with especially large samples (Nylund-Gibson and Choi, 2018). Entropy was highest for the five-and six-profile solutions.

**Table 2:** Model fit statistics for latent profile analysis, by sample and profile solution.

No.	Params.	LL	BIC	SABIC	CAIC	AWE	VLMR -LRT *p*	Entropy	BF	cmP
1	6	−77461.07	154980.42	154961.35	154983.54	154986.54	--	--	0.00	0.000
2	10	−74089.80	148276.74	148244.96	148281.94	148286.94	0.000	0.711	0.00	0.000
3	14	−72344.91	144825.82	144781.33	144833.10	144840.10	0.000	0.718	0.00	0.000
4	18	−71837.65	143850.15	143792.94	143859.51	143868.51	0.003	0.899	0.00	0.000
5	22	−67292.16	134798.03	134728.11	134809.48	134820.48	0.000	0.999	0.00	0.000
6	26	−54560.66	109373.88	109291.26	109387.41	109400.41	0.071	0.999	0.00	0.000
7	30	−54492.41	109276.24	109180.90	109291.85	109306.85	0.497	0.949	0.00	0.000
8	34	−54342.94	109016.15	108908.10	109033.84	109050.84	0.496	0.941	0.00	0.000
9	38	−54222.35	108813.83	108693.07	108833.61	108852.61	0.495	0.881	0.00	0.000
10	42	−54194.24	108796.47	108662.99	108818.32	108839.32	0.499	0.875	--	1.000

*N* = 16,547. LL, log likelihood. BIC, Schwarz’s Bayes information criterion. SABIC, sample size-adjusted Bayes information criterion. CAIC, consistent akaike information criterion. AWE, average weight extracted. VLMR-LRT, Vuong-Lo-Mendell-Rubin likelihood ratio test. BF, Bayes Factor. cmP, cumulative model probability.

**Figure 1:**
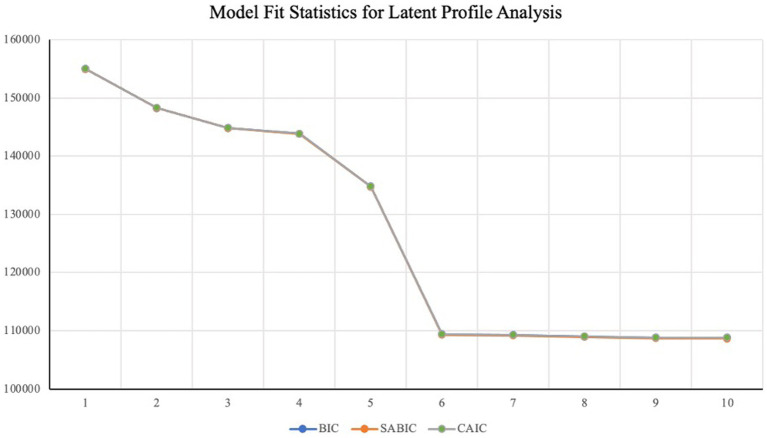
Model fit statistics for latent profile analysis.

Figure 1 presents a visual summary of BIC, SABIC, and CAIC statistics for each of the profile solutions from 1 to 10. The figure shows a significant drop in all three values moving from five to six profiles, and then exhibits diminishing returns thereafter, with little improvement for the 6-to-10 profile solutions. Thus, given the alignment between the VLMR-LRT test results and the visual inspection of the trend in fit statistics, we chose to move forward with the five-profile solution.

### 4.2. Further articulating learning profiles

**Figure 2:**
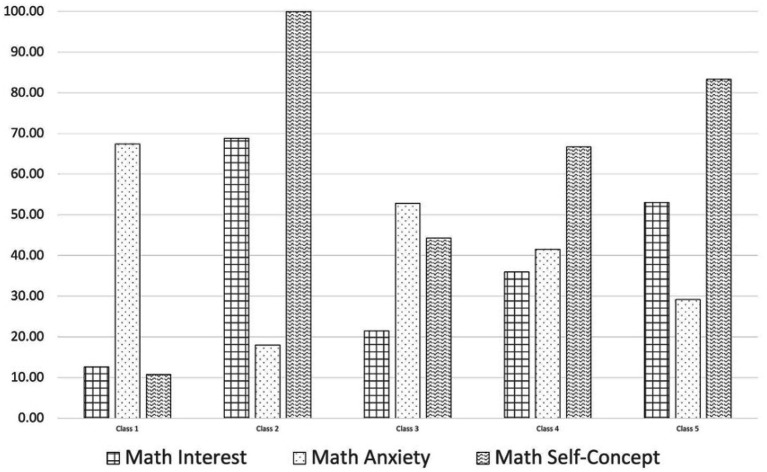
Conditional means of math interest, math anxiety, and math self-concept for the preferred five class solution.

Having chosen the 5-profile solution based on the available fit evidence, we next created a conditional means plot to visualize the relative shapes and levels of each profile. Conditional means, adjusting for the classification error for each student, were generated for math self-concept, anxiety, and interest for each of the 5 profiles (see Figure 2). The POMP transformation was applied to allow for comparisons between items measured on different scales.

Table 3 includes descriptive statistics by profile, along with their sample sizes in parentheses.

**Table 3:** Profile-specific descriptive statistics for latent profile analysis (LPA) indicators.

Variable	Estimate	SE	*p*
Latent profile 1 (*n* = 549)			
Math interest	1.505	0.053	<0.001
Math anxiety	3.697	0.080	<0.001
Math self-concept	1.645	0.030	<0.001
Latent profile 2 (*n* = 1,637)			
Math interest	3.753	0.048	<0.001
Math anxiety	1.720	0.036	<0.001
Math self-concept	7.000	0.000	<0.001
Latent profile 3 (*n* = 2,619)			
Math interest	1.858	0.028	<0.001
Math anxiety	3.113	0.034	<0.001
Math self-concept	3.656	0.015	<0.001
Latent profile 4 (*n* = 6,065)			
Math interest	2.439	0.018	<0.001
Math anxiety	2.663	0.019	<0.001
Math self-concept	5.000	0.000	<0.001
Latent profile 5 (*n* = 5,677)			
Math interest	3.122	0.018	<0.001
Math anxiety	2.168	0.018	<0.001
Math self-concept	6.000	0.000	<0.001

*N* = 16,547.

Profiles 1 (3% of sample) and 3 (16% of sample) include students that tended to have higher levels of anxiety and lower levels of self-concept and interest. Profile 1 demonstrated the highest average levels of anxiety (more than 3.50 out of 5 scale points), and profile 3 had the second highest at 3.1 out of 5 points. Compared to Profile 3, Profile 1 had lower levels of both self-concept and interest. Profiles 2 (10% of sample) and 5 (34%) are characterized by lower levels of anxiety and high levels of self-concept and interest. Profile 2 had the lowest level of anxiety (1.70 out of 5) but differing slightly levels of interest (3.70 out of 5) and self-concept (7 out of 7) with profile members having the highest self-concept possible. Students in profile 5 had slightly lower interest (3.10 out of 5) and self-concept (6 out of 7), and higher anxiety than profile 2 (2.20 out of 5). This aligns with prior literature that higher value and control appraisal have negative associations with math anxiety. Profile 4 (37% of sample) represents the largest group in our sample and is characterized by students that had average math interest (2.40 out of 5) with slightly higher anxiety (2.70 out of 5), and higher self-concept (5 out of 7).

### 4.3. Learning profiles and distal outcomes

We next analyzed the extent to which profile membership might be associated with differential responses on distal variables, including challenge seeking behavior, prior math achievement, and academic stress. Results of this analysis are available in Table 4. Similar to the previous section, we present the conditional mean (or proportion) associated with each outcome. In footnotes, we include all significant pairwise profile differences.

**Table 4:** Estimated profile means and differences in distal outcomes pairwise profile differences on distal variables.

Challenge seeking behavior				
			Mean	S.E.
Profile 1			0.246	0.026
Profile 2			0.593	0.019
Profile 3			0.334	0.015
Profile 4			0.403	0.009
Profile 5			0.487	0.010
Overall			Chi-Square	*p*
			207.026	<0.001
Profile 1	vs.	2	116.426	<0.001
Profile 1	vs.	3	8.729	<0.01
Profile 1	vs.	4	32.844	<0.001
Profile 1	vs.	5	77.240	<0.001
Profile 2	vs.	3	111.615	<0.001
Profile 2	vs.	4	78.747	<0.001
Profile 2	vs.	5	24.093	<0.001
Profile 3	vs.	4	15.021	<0.001
Profile 3	vs.	5	73.470	<0.001
Profile 4	vs.	5	39.996	<0.001
**Academic stress**				
			**Mean**	**S.E.**
Profile 1			3.656	0.068
Profile 2			2.763	0.051
Profile 3			3.392	0.038
Profile 4			3.126	0.021
Profile 5			2.920	0.022
Overall			Chi-Square	*p*
			235.623	<0.001
Profile 1	vs.	2	109.791	<0.001
Profile 1	vs.	3	11.431	<0.001
Profile 1	vs.	4	54.911	<0.001
Profile 1	vs.	5	104.757	<0.001
Profile 2	vs.	3	98.719	<0.001
Profile 2	vs.	4	43.846	<0.001
Profile	vs.	5	8.022	<0.01
Profile 3	vs.	4	38.010	<0.001
Profile 3	vs.	5	116.335	<0.001
Profile 4	vs.	5	46.423	<0.001
**Prior math GPA**				
			**Mean**	**S.E.**
Profile 1			2.192	0.085
Profile 2			3.388	0.045
Profile 3			2.396	0.033
Profile 4			2.752	0.022
Profile 5			3.181	0.023
Overall			Chi-square	*p*
			620.458	<0.001
Profile 1	vs.	2	153.840	<0.001
Profile 1	vs.	3	4.965	<0.05
Profile 1	vs.	4	40.338	<0.001
Profile 1	vs.	5	125.518	<0.001
Profile 2	vs.	3	317.985	<0.001
Profile 2	vs.	4	161.106	<0.001
Profile 2	vs.	5	16.959	<0.001
Profile 3	vs.	4	80.389	<0.001
Profile 3	vs.	5	387.215	<0.001
Profile 4	vs.	5	182.827	<0.001

*N* = 16,547.

#### 4.3.1. Challenge-seeking behavior

Overall, the percentage of students who attempted a challenging math problem ranged from a minimum of 25% in Profile 1 to a maximum of 59% in Profile 2. In a variable-centered analysis, we might hypothesize that challenge-seeking is positively associated with self-concept and interest, and negatively associated with math anxiety. If this holds within-person, then we might expect that students in profiles with high self-concept and low anxiety might demonstrate higher levels of challenge seeking behavior. Our analysis confirms this. For example, Profiles 2 and 5 both have high self-concept and high interest. Although they exhibit slightly different levels of challenge seeking, 59% in Profile 2 and 49% in Profile 5, they have the highest levels of challenge seeking amongst all the profiles. We also found that Profile 4, a profile with medium anxiety, had the next highest levels of challenge seeking, at 40%.

#### 4.3.2. Math prior achievement

Overall, 8th grade math GPA ranged from a minimum conditional mean of 2.19 (out of 4.30) in Profile 1 to a maximum conditional mean of 3.39 in Profile 2. We would expect that math prior achievement would likely relate positively to self-concept and interest, and negatively to anxiety. Thus, we expected that Profiles 2 and 5 might have the highest math prior achievement. Our results confirm this, as Profile 2 exhibited the highest mean prior achievement (this profile also had the highest self-concept and interest). Profile 5 had the second-highest prior achievement.

#### 4.3.3. Academic stress

Overall, academic stress ranged from a minimum conditional mean of 2.76 (out of 5) in Profile 2 to a maximum conditional mean of 3.66 in Profile 1. This is less variation than was observed for math anxiety, which ranged from a minimum of 1.70 in Profile 1 to a maximum of 3.70 in Profile 2. In theory, academic stress should map most closely onto math anxiety, so we hypothesized that students in Profiles 1 and 3 should also have the highest academic stress. Our results confirm this. We found that the profile with the highest academic stress, Profile 1, had high levels of anxiety. Profile 3, the next highest in academic stress, was high for anxiety as well.

## 5. Discussion

Using an exploratory approach to latent profile analysis and including math anxiety, self-concept, and math interest as indicators, we found significant evidence that extends on previous research on students’ math learning, emotions, and motivation by investigating control-value profiles of students’ math motivation and emotions in a nationally representative sample of more than 16,000 ninth grade students in the United States. We also examined the extent to which student profile membership predicts academic stress and challenge-seeking behaviors, and the relationship between student profile membership and prior math achievement.

Results call for the need to look beyond linear relations among these complex, multifaceted constructs. Several prior studies that analyze both control and value appraisal have found results that do not strictly align with the framework that value appraisal has negative associations with math anxiety (Ganley and McGraw, 2016; Malanchini et al., 2020). Pekrun’s control-value theory (Pekrun, 2006) and much prior research (Ashcraft and Kirk, 2001; Park et al., 2014; Ramirez et al., 2018) find an inverse relationship between math anxiety and math achievement. This is consistent with all profiles found in the NSLM sample. Profiles 1 and 3 included students with lower levels of prior math achievement who reported higher levels of math anxiety. While for the students in profiles 2 and 5 higher math achievement was paired with low math anxiety. Profile 4, the largest profile identified in this study, illustrates the contribution of a within-person approach to examining these constructs. Composing more than 37% of the total sample, and demonstrating medium levels of interest, high levels of self-concept, and medium levels of anxiety, this profile exhibits patterns that are not easily identified in variable-centered analyses testing C-VTAE. This specific profile could well be overlooked or unnoticed in a variable-centered framework.

Math interest is consistently found to have a positive association with achievement (Köller et al., 2001). Interventions that target increasing interest such as utility-value interventions are found to minimize achievement gaps (Harackiewicz et al., 2016). In our study, the two profiles with the highest levels of math interest, Profiles 2 and 5 in Figure 2, also tended to have the highest prior math GPAs. We also found that math interest and math self-concept appeared to mirror each other closely, with profiles that exhibited higher levels of interest also exhibiting higher levels of self-concept, and vice versa. This pattern aligns with results from Wang et al. (2018), which found that students’ math interest and math self-perception (a different but related concept than self-concept), also were very closely related across all eight of their latent profiles.

However, the largest profile in our sample did not exhibit a clear alignment with the framework. Although in prior research, when control and value appraisal are studied together, the negative association between math self-concept and anxiety is stronger when the student’s math value is perceived as higher. This group showed lower math interest than self-concept, which may explain the higher math anxiety. Profiles aligned with theory in predicting whether students would attempt a challenging problem. The differences noted here were the anxiety levels between the two groups. We expected self-concept to relate positively to interest and negatively to anxiety, which aligns with the profiles with highest math self-concept. The research literature demonstrates that math anxiety can have conflicting, and at times unexpected, relationships with students’ math interest and self-concept. For example, as Wang et al. (2015) noted, anxiety can have nonlinear relationships with achievement, often dependent upon students’ motivation; Abín et al. (2020) found using variable-centered methods that the contribution of math anxiety to math achievement may be minimal after accounting for students’ intrinsic interest and utility value. Thus, it is not surprising that a significant portion of the sample demonstrated patterns that may not strictly adhere to theory.

This work furthers the research related to STEM disciplines in that achievement is not the only factor contributing to STEM learning, but psychological and socio-emotional factors have an impact as well. This theme was a key component of a recent report by the National Academies of Sciences, Engineering, and Medicine (2019), and reinforced by Wang et al.’s (2018) latent profile analysis of high school students in Italy, as was well as by Malanchini et al. (2020), which incorporated genetic factors in addition to math motivation, anxiety, and performance. This work also highlights the importance of taking a group centered approach when analyzing these constructs. Similar to Wang et al. (2018), we find distinct multidimensional profiles that characterize students’ interpersonal variability in math anxiety, interest, and self-concept, and that these largely align with students’ performance in mathematics. This research contributes to identifying learning profiles to better understand school and classroom factors and how they can shape achievement, behaviors, and beliefs.

### 5.1. Limitations and future directions

This work has several limitations that are important to emphasize. First, the profile indicators we used here for student math anxiety and self-concept were responses to a single item on the NSLM survey. We acknowledge that single items are typically not ideal for measuring psychological constructs. However, in this case, given that the NSLM was not primarily designed to assess the constructs we use here (and rather to focus on learning mindsets), we believe that the advantage of having a large, generalizable sample of responses outweighs the drawbacks of only having single item responses. In some cases, single-item measures can be appropriate to measure psychological constructs provided that the item is clearly written, with a clear object and attribute (Bergkvist, 2015). We believe this is the case for the items we use. Given the data collection timeline for NSLM, we used 8th grade math GPA as the indicator of school math performance. Ideally, our measure of performance would come directly from 9th grade, however 8th grade math GPA is likely to strongly correlate with 9th grade math performance. Finally, we want to caution readers that our analysis does not allow for causal inferences, as students are not randomly assigned to classrooms, nor is profile membership exogenous from other unobserved measures that may not be included in this dataset. In addition, all of these measures (except prior math GPA) were collected in the same online session. Therefore, any relationships between profile membership and other student variables should not be seen as causal.

This work also suggests a number of future directions in this area that might be beneficial. For example, it would be important to explore potential differences in profile composition related to gender, minoritized racial and ethnic groups, and socioeconomic status, especially given the longstanding inequities and underrepresentation in STEM (National Science Board, 2021). It is also important to consider a longitudinal perspective, perhaps examining change in student profile membership over time *via* latent transition analysis or a similar approach. These profiles are likely not static, and as high school students continue to develop there could be both changes in profile membership as well as potential changes in the pattern and structure of the profiles overall. Wang et al. (2021) have begun this work by following middle and high school students over time and mapping the co-development of math anxiety along with control and value appraisals, but more evidence is needed to fully describe how this change process may occur.

### 5.2. Conclusion

This work builds on and extends prior research on students’ math motivation and emotions in the transition to high school. We found a variety of complex student profiles which conform to many of the tenets of C-VTAE. However, we also found that our largest profile, comprising nearly 40% of the total sample, displayed a pattern of anxiety, interest, and self-concept that did not clearly separate as might be expected. It is possible that this group is made up of students who are relatively average or typical in their levels of these indicators, and as such do not display the separation seen in the other profiles. Nonetheless, this finding is interesting, given the size of the profile and suggests that for many students, the hypothesized relationship between these three constructs may not be as clear. We hope that this work can further elucidate the tenets of C-VTAE in a within-person context while also contributing to a more nuanced and complex understanding of the interplay between student motivation, emotion, and achievement in mathematics.

## Data availability statement

The datasets presented in this study can be found in online repositories. The names of the repository/repositories and accession number(s) can be found in the article/supplementary material.

## Ethics statement

The studies involving human participants were reviewed and approved by Institutional Review Board, University of Texas at Austin. Written informed consent from the participants’ legal guardian/next of kin was not required to participate in this study in accordance with the national legislation and the institutional requirements.

## Author contributions

MB led all aspects of the study, including conceptualization, data analysis and interpretation, manuscript drafting, revising, and editing. ER assisted with results interpretation, manuscript drafting, revising, and editing. NS assisted with manuscript revising and editing. EE assisted with the initial draft of the manuscript. All authors contributed to the article and approved the submitted version.

## Funding

Research reported in this summary was supported by the National Study of Learning Mindsets Early Career Fellowship with funding provided by the Bezos Family Foundation. The University of Texas at Austin receives core support from the National Institute of Child Health and Human Development under the award number 5R24 HD042849. This publication uses data from the National Study of Learning Mindsets (doi: 10.3886/ICPSR37353.v1; PI: D. Yeager; Co-Is: R. Crosnoe, C. Dweck, C. Muller, B. Schneider, and G. Walton), which was made possible through methods and data systems created by the Project for Education Research That Scales (PERTS), data collection carried out by ICF International, meetings hosted by the Mindset Scholars Network at the Center for Advanced Study in the Behavioral Sciences at Stanford University, assistance from C. Hulleman, R. Ferguson, M. Shankar, T. Brock, C. Romero, D. Paunesku, C. Macrander, T. Wilson, E. Konar, M. Weiss, E. Tipton, and A. Duckworth, and funding from the Raikes Foundation, the William T. Grant Foundation, the Spencer Foundation, the Bezos Family Foundation, the Character Lab, the Houston Endowment, the National Institutes of Health under award number R01HD084772-01, the National Science Foundation under grant number 1761179, Angela Duckworth (personal gift), and the President and Dean of Humanities and Social Sciences at Stanford University. The content is solely the responsibility of the authors and does not necessarily represent the official views of the Bezos Family Foundation, the Mindset Scholars Network, The University of Texas at Austin Population Research Center, the National Institutes of Health, the National Science Foundation, or other funders.

## Conflict of interest

The authors declare that the research was conducted in the absence of any commercial or financial relationships that could be construed as a potential conflict of interest.

## Publisher’s note

All claims expressed in this article are solely those of the authors and do not necessarily represent those of their affiliated organizations, or those of the publisher, the editors and the reviewers. Any product that may be evaluated in this article, or claim that may be made by its manufacturer, is not guaranteed or endorsed by the publisher.

## References

[ref1] Abell S. K. (2007). “Research on science teacher knowledge” in Handbook of Research on Science Education. eds. Abell S. K. Lederman N. G. (Mahweh: Lawrence Erlbaum), 1105–1149.

[ref2] Abín A. Núñez J. C. Rodríguez C. Cueli M. García T. Rosário P. (2020). Predicting mathematics achievement in secondary education: the role of cognitive, motivational, and emotional variables. Front. Psychol. 11:876. doi: 10.3389/fpsyg.2020.00876, PMID: 32528351 PMC7264990

[ref3] Ahmed W. Minnaert A. Kuyper H. van der Werf G. (2012). Reciprocal relationships between math self-concept and math anxiety. Learn. Individ. Differ. 22, 385–389. doi: 10.1016/j.lindif.2011.12.004

[ref4] Ainley M. (2007). “Chapter 9-being and feeling interested: transient state, mood, and disposition” in Emotion in Education. eds. Schutz P. A. Pekrun R. (Burlington: Academic Press), 147–163.

[ref5] Ainley M. Hidi S. Berndorff D. (2002). Interest, learning, and the psychological processes that mediate their relationship. J. Educ. Psychol. 94, 545–561. doi: 10.1037//0022-0663.94.3.545

[ref6] Alexander P. A. (2003). The development of expertise: the journey from acclimation to proficiency. Educ. Res. 32, 10–14. doi: 10.3102/0013189x032008010

[ref7] Alexander P. A. Murphy P. K. (1998). Profiling the differences in students’ knowledge, interest, and strategic processing. J. Educ. Psychol. 90, 435–447. doi: 10.1037/0022-0663.90.3.435

[ref8] Alexander P. A. Murphy P. K. Woods B. S. Duhon K. E. Parker D. (1997). College instruction and concomitant changes in students’ knowledge, interest, and strategy use: a study of domain learning. Contemp. Educ. Psychol. 22, 125–146. doi: 10.1006/ceps.1997.0927

[ref9] Ashcraft M. H. Kirk E. P. (2001). The relationships among working memory, math anxiety, and performance. J. Exp. Psychol. Gen. 130, 224–237. doi: 10.1037/0096-3445.130.2.224 11409101

[ref10] Bae C. L. DeBusk-Lane M. (2018). Motivation belief profiles in science: links to classroom goal structures and achievement. Learn. Individ. Differ. 67, 91–104. doi: 10.1016/j.lindif.2018.08.003

[ref11] Bandura A. (1986). Social Foundations of Thought and Action. Englewood Cliffs, NJ: Prentice-Hall, Inc.

[ref12] Banfield J. D. Raftery A. E. (1993). Model-based Gaussian and non-Gaussian clustering. Biometrics 49, 803–821. doi: 10.2307/2532201

[ref13] Bergkvist L. (2015). Appropriate use of single-item measures is here to stay. Mark. Lett. 26, 245–255. doi: 10.1007/s11002-014-9325-y

[ref14] Bergkvist L. Rossiter J. R. (2007). The predictive validity of multiple-item versus single-item measures of the same constructs. J. Mark. Res. 44, 175–184. doi: 10.1509/jmkr.44.2.175

[ref15] Berlin K. S. Williams N. A. Parra G. R. (2014). An introduction to latent variable mixture modeling (part 1): overview and cross-sectional latent class and latent profile analyses. J. Pediatr. Psychol. 39, 174–187. doi: 10.1093/jpepsy/jst084, PMID: 24277769

[ref16] Bieg M. Goetz T. Hubbard K. (2013). Can I master it and does it matter? An intraindividual analysis on control–value antecedents of trait and state academic emotions. Learn. Individ. Differ. 28, 102–108. doi: 10.1016/j.lindif.2013.09.006

[ref17] Boekaerts M. Pekrun R. (2015). “Emotions and emotion regulation in academic settings” in Handbook of Educational Psychology, Vol. 3. eds. L. Corno and E. M. Anderman (London: Routledge), 76–90.

[ref18] Bolck A. Croon M. Hagenaars J. (2004). Estimating latent structure models with categorical variables: one-step versus three-step estimators. Polit. Anal. 12, 3–27. doi: 10.1093/pan/mph001

[ref19] Bozdogan H. (1987). Model selection and Akaike’s information criterion (AIC): the general theory and its analytical extensions. Psychometrika 52, 345–370. doi: 10.1007/BF02294361

[ref20] Chen J. A. Usher E. L. (2013). Profiles of the sources of science self-efficacy. Learn. Individ. Differ. 24, 11–21. doi: 10.1016/j.lindif.2012.11.002

[ref21] Cohen P. Cohen J. Aiken L. S. West S. G. (1999). The problem of units and the circumstance for POMP. Multivar. Behav. Res. 34, 315–346. doi: 10.1207/S15327906MBR3403_2

[ref22] Everitt B. S. (1979). Unresolved problems in cluster analysis. Biometrics 35, 169–181. doi: 10.2307/2529943

[ref1003] Finch W. H. French B. F. (2014). Multilevel Latent Class Analysis: Parametric and Nonparametric Models. J. Exper. Educ. 82, 307–333. doi: 10.1080/00220973.2013.813361

[ref23] Fredricks J. A. Eccles J. S. (2002). Children’s competence and value beliefs from childhood through adolescence: growth trajectories in two male-sex-typed domains. Dev. Psychol. 38, 519–533. doi: 10.1037/0012-1649.38.4.519, PMID: 12090482

[ref24] Frenzel A. C. Goetz T. Lüdtke O. Pekrun R. Sutton R. E. (2009). Emotional transmission in the classroom: exploring the relationship between teacher and student enjoyment. J. Educ. Psychol. 101, 705–716. doi: 10.1037/a0014695

[ref25] Frenzel A. C. Goetz T. Pekrun R. Watt H. M. G. (2010). Development of mathematics interest in adolescence: influences of gender, family, and school context: DEVELOPMENT OF MATHEMATICS INTEREST IN ADOLESCENCE. J. Res. Adolesc. 20, 507–537. doi: 10.1111/j.1532-7795.2010.00645.x

[ref26] Ganley C. M. McGraw A. L. (2016). The development and validation of a revised version of the math anxiety scale for young children. Front. Psychol. 7:1181. doi: 10.3389/fpsyg.2016.01181, PMID: 27605917 PMC4995220

[ref27] Gogol K. Brunner M. Goetz T. Martin R. Ugen S. Keller U.. (2014). “My questionnaire is too long!” the assessments of motivational-affective constructs with three-item and single-item measures. Contemp. Educ. Psychol. 39, 188–205. doi: 10.1016/j.cedpsych.2014.04.002

[ref28] Gopalan M. Tipton E. (2018). Is the National Study of learning mindsets nationally-representative? Available at: https://osf.io/dvmr7/download?format=pdf.

[ref29] Gross J. J. (2015). Emotion regulation: current status and future prospects. Psychol. Inq. 26, 1–26. doi: 10.1080/1047840X.2014.940781

[ref30] Harackiewicz J. M. Smith J. L. Priniski S. J. (2016). Interest matters: the importance of promoting interest in education. Policy Insights Behav. Brain Sci. 3, 220–227. doi: 10.1177/2372732216655542, PMID: 29520371 PMC5839644

[ref31] Hart S. A. Logan J. A. R. Thompson L. Kovas Y. McLoughlin G. Petrill S. A. (2016). A latent profile analysis of math achievement, numerosity, and math anxiety in twins. J. Educ. Psychol. 108, 181–193. doi: 10.1037/edu0000045, PMID: 26957650 PMC4779361

[ref32] Hidi S. Renninger K. A. (2006). The four-phase model of interest development. Educ. Psychol. 41, 111–127. doi: 10.1207/s15326985ep4102_4

[ref33] Huber P. J. The behavior of maximum likelihood estimates under nonstandard conditions. In: Proceedings of the fifth Berkeley symposium on mathematical statistics and probability (University of California Press); 1967. p. 221–233. Available at: https://books.google.com/books?hl=en&lr=&id=IC4Ku_7dBFUC&oi=fnd&pg=PA221&dq=huber+1967&ots=nOXiJZK8vK&sig=dMcDej2_XAtWFkf33GRj3h0N_HU.

[ref34] Jovanović V. Lazić M. (2020). Is longer always better? A comparison of the validity of single-item versus multiple-item measures of life satisfaction. Appl. Res. Qual. Life 15, 675–692. doi: 10.1007/s11482-018-9680-6

[ref35] Justicia-Galiano M. J. Martín-Puga M. E. Linares R. Pelegrina S. (2017). Math anxiety and math performance in children: the mediating roles of working memory and math self-concept. Br. J. Educ. Psychol. 87, 573–589. PMID: 28561304 10.1111/bjep.12165

[ref36] Kaskens J. Segers E. Goei S. L. van Luit J. E. H. Verhoeven L. (2020). Impact of Children’s math self-concept, math self-efficacy, math anxiety, and teacher competencies on math development. Teach. Teach. Educ. 94:103096. doi: 10.1016/j.tate.2020.103096

[ref37] Köller O. Baumert J. Schnabel K. (2001). Does interest matter? The relationship between academic interest and achievement in mathematics. J. Res. Math. Educ. 32, 448–470. doi: 10.2307/749801

[ref38] Kosovich J. J. Hulleman C. S. Phelps J. Lee M. (2019). Improving algebra success with a utility-value intervention. J. Dev. Educ. 42, 2–10.

[ref39] Lo Y. Mendell N. R. Rubin D. B. (2001). Testing the number of components in a normal mixture. Biometrika 88, 767–778. doi: 10.1093/biomet/88.3.767

[ref40] Lyons I. M. Beilock S. L. (2012). When math hurts: math anxiety predicts pain network activation in anticipation of doing math. PLoS One 7:e48076. doi: 10.1371/journal.pone.0048076, PMID: 23118929 PMC3485285

[ref41] Ma X. (1999). A meta-analysis of the relationship between anxiety toward mathematics and achievement in mathematics. J. Res. Math. Educ. 30:520. doi: 10.2307/749772

[ref42] Malanchini M. Rimfeld K. Wang Z. Petrill S. A. Tucker-Drob E. M. Plomin R.. (2020). Genetic factors underlie the association between anxiety, attitudes and performance in mathematics. Transl. Psychiatry 10:12. doi: 10.1038/s41398-020-0711-3, PMID: 32066693 PMC7026074

[ref43] Marsh H. W. Craven R. (1996). “Chapter 6—academic self-concept: beyond the dustbowl” in Handbook of Classroom Assessment. ed. Phye G. D. (San Diego: Academic Press), 131–198.

[ref44] Marsh H. W. Ellis L. A. Parada R. H. Richards G. Heubeck B. G. (2005). A short version of the self description questionnaire II: operationalizing criteria for short-form evaluation with new applications of confirmatory factor analyses. Psychol. Assess. 17, 81–102. doi: 10.1037/1040-3590.17.1.81, PMID: 15769230

[ref45] Marsh H. W. Martin A. J. (2010). Academic self-concept and academic achievement: relations and causal ordering. Br. J. Educ. Psychol. 81, 59–77. doi: 10.1348/000709910X503501, PMID: 21391964

[ref46] Masyn K. E. (2013). “Latent class analysis and finite mixture modeling” in The Oxford handbook of quantitative methods Oxford library of psychology. ed. Little T. D. (Oxford: Oxford University Press), 551–611.

[ref47] McLachlan G. J. Lee S. X. Rathnayake S. I. (2019). Finite Mixture Models. Ann. Rev. Stat. Appl. 6, 355–378. doi: 10.1146/annurev-statistics-031017-100325

[ref48] Muis K. R. Chevrier M. Singh C. A. (2018). The role of epistemic emotions in personal epistemology and self-regulated learning. Educ. Psychol. 53, 165–184. doi: 10.1080/00461520.2017.1421465

[ref49] Murphy P. K. Alexander P. A. (2002). What counts? The predictive powers of subject-matter knowledge, strategic processing, and interest in domain-specific performance. J. Exp. Educ. 70, 197–214. doi: 10.1080/00220970209599506

[ref50] Namkung J. M. Peng P. Lin X. (2019). The relation between mathematics anxiety and mathematics performance among school-aged students: a meta-analysis. Rev. Educ. Res. 89, 459–496. doi: 10.3102/0034654319843494

[ref51] National Academies of Sciences, Engineering, and Medicine, National Academy of Engineering, Division of Behavioral and Social Sciences and Education, Board on Science Education, and Committee on Science Investigations and Engineering Design Experiences in Grades 6-12 (2019). Science and Engineering for Grades 6-12: Investigation and Design at the Center. Washington, DC: National Academies Press.

[ref52] National Research Council, Division of Behavioral and Social Sciences and Education, Board on Science Education, and Committee on a Conceptual Framework for New K-12 Science Education Standards (2012). A Framework for K-12 Science Education: Practices, Crosscutting Concepts, and Core Ideas. Washington, DC: National Academies Press.

[ref53] National Science Board. (2021). Science and engineering indicators 2021. National Science Foundation. August. Available at: https://ncses.nsf.gov/pubs/nsb20212/.

[ref54] Nie Y. Lau S. Liau A. K. (2011). Role of academic self-efficacy in moderating the relation between task importance and test anxiety. Learn. Individ. Differ. 21, 736–741. doi: 10.1016/j.lindif.2011.09.005

[ref55] Nylund K. L. Asparouhov T. (2007). Deciding on the number of classes in latent class analysis and growth mixture modeling: A Monte Carlo simulation study. Struct. Equ. Model. doi: 10.1080/10705510701575396

[ref56] Nylund-Gibson K. Choi A. Y. (2018). Ten frequently asked questions about latent class analysis. Transl. Issues Psychol. 4, 440–461. doi: 10.1037/tps0000176

[ref57] Nylund-Gibson K. Grimm R. P. Masyn K. E. (2019). Prediction from latent classes: a demonstration of different approaches to include distal outcomes in mixture models. Struct. Equ. Model. 26, 967–985. doi: 10.1080/10705511.2019.1590146

[ref58] Orbach L. Fritz A. (2022). A latent profile analysis of math anxiety and core beliefs toward mathematics among children. Ann. N. Y. Acad. Sci. 1509, 130–144. doi: 10.1111/nyas.14720, PMID: 34791691

[ref59] Park D. Ramirez G. Beilock S. L. (2014). The role of expressive writing in math anxiety. J. Exp. Psychol. Appl. 20, 103–111. doi: 10.1037/xap0000013, PMID: 24708352

[ref60] Parker P. D. Marsh H. W. Ciarrochi J. Marshall S. Abduljabbar A. S. (2013). Juxtaposing math self-efficacy and self-concept as predictors of long-term achievement outcomes. Educ. Psychol. 34, 29–48. doi: 10.1080/01443410.2013.797339

[ref61] Patall E. A. Steingut R. R. Vasquez A. C. Trimble S. S. Pituch K. A. Freeman J. L. (2018). Daily autonomy supporting or thwarting and students’ motivation and engagement in the high school science classroom. J. Educ. Psychol. 110, 269–288. doi: 10.1037/edu0000214

[ref62] Peixoto F. Sanches C. Mata L. Monteiro V. (2017). How do you feel about math? relationships between competence and value appraisals, achievement emotions and academic achievement. Eur. J. Psychol. Educ. 32, 385–405. doi: 10.1007/s10212-016-0299-4

[ref63] Pekrun R. (2006). The control-value theory of achievement emotions: assumptions, corollaries, and implications for educational research and practice. Educ. Psychol. Rev. 18, 315–341. doi: 10.1007/s10648-006-9029-9

[ref64] Pekrun R. Frenzel A. C. Goetz T. Perry R. P. (2007). “The control-value theory of achievement emotions: an integrative approach to emotions in education” in Emotion in education. eds. Schutz P. A. Pekrun R. (Burlington: Academic Press), 13–36.

[ref65] Pekrun R. Goetz T. Frenzel A. C. Barchfeld P. Perry R. P. (2011). Measuring emotions in students’ learning and performance: the achievement emotions questionnaire (AEQ). Contemp. Educ. Psychol. 36, 36–48. doi: 10.1016/j.cedpsych.2010.10.002

[ref66] Pekrun R. Goetz T. Titz W. Perry R. P. (2002). Academic emotions in students’ self-regulated learning and achievement: a program of qualitative and quantitative research. Educ. Psychol. 37, 91–105. doi: 10.1207/S15326985EP3702_4

[ref67] Pekrun R. Lichtenfeld S. Marsh H. W. Murayama K. Goetz T. (2017). Achievement emotions and academic performance: longitudinal models of reciprocal effects. Child Dev. 88, 1653–1670. doi: 10.1111/cdev.12704, PMID: 28176309

[ref68] Ramirez G. Hooper S. Y. Kersting N. B. Ferguson R. Yeager D. (2018). Teacher math anxiety relates to adolescent students. Math achievement. AERA Open 4:233285841875605. doi: 10.1177/2332858418756052, PMID: 31069247 PMC6502250

[ref69] Ramirez G. Shaw S. T. Maloney E. A. (2018). Math anxiety: past research, promising interventions, and a new interpretation framework. Educ. Psychol. 53, 145–164. doi: 10.1080/00461520.2018.1447384

[ref70] Satorra A. Bentler P. M. (2001). A scaled difference chi-square test statistic for moment structure analysis. Psychometrika 66, 507–514. doi: 10.1007/BF02296192 PMC2905175 20640194

[ref71] Schiefele U. (1999). Interest and learning from text. Sci. Stud. Read. 3, 257–279. doi: 10.1207/s1532799xssr0303_4

[ref72] Schiefele U. (2001). “The role of interest in motivation and learning,’’ in Intelligence and Personality: Bridging the Gap in Theory and Measurement (1st ed.). eds. J. M. Collis, S. J. Messick and U. Schiefele (New York: Psychology Press), 163–194.

[ref73] Schiefele U. Krapp A. Winteler A. (1992). “Interest as a predictor of academic achievement: a meta-analysis of research” in The Role of Interest in Learning and Development. ed. Renninger K. A. (Hillsdale, NJ, US: Lawrence Erlbaum Associates, Inc, xiv), 183–212.

[ref74] Schwarz G. (1978). Estimating the dimension of a model. Ann. Stat. 6, 461–464. doi: 10.1214/aos/1176344136

[ref75] Sclove S. L. (1987). Application of model-selection criteria to some problems in multivariate analysis. Psychometrika 52, 333–343. doi: 10.1007/BF02294360

[ref76] Shavelson R. J. Hubner J. J. Stanton G. C. (1976). Self-concept: validation of construct interpretations. Rev. Educ. Res. 46, 407–441. doi: 10.3102/00346543046003407

[ref77] Timmerman H. L. Toll S. W. M. Van Luit J. E. H. (2017). The relation between math self-concept, test and math anxiety, achievement motivation and math achievement in 12 to 14-year-old typically developing adolescents. Psychol. Soc. Educ. 9, 89–103. doi: 10.25115/psye.v9i1.465

[ref1001] Tipton E. Yeager D. S. Iachan R. Schneider B. (2019). “Designing probability samples to study treatment effect heterogeneity,’’ in Experimental Methods in Survey Research. eds. P. Lavrakas, M. Traugott, C. Kennedy, A. Holbrook, E. de Leeuw, and B. West (Wiley), 435–456.

[ref78] Usher E. L. Pajares F. (2008). Sources of self-efficacy in school: critical review of the literature and future directions. Rev. Educ. Res. 78, 751–796. doi: 10.3102/0034654308321456

[ref79] Wagenmakers E.-J. (2007). A practical solution to the pervasive problems of p values. Psychon. Bull. Rev. 14, 779–804. doi: 10.3758/bf03194105 18087943

[ref80] Wang Z. Borriello G. A. Oh W. Lukowski S. Malanchini M. (2021). Co-development of math anxiety, math self-concept, and math value in adolescence: the roles of parents and math teachers. Contemp. Educ. Psychol. 67:102016. doi: 10.1016/j.cedpsych.2021.102016

[ref81] Wang Y.-L. Liang J.-C. Lin C.-Y. Tsai C.-C. (2017). Identifying Taiwanese junior-high school students’ mathematics learning profiles and their roles in mathematics learning self-efficacy and academic performance. Learn. Individ. Differ. 54, 92–101. doi: 10.1016/j.lindif.2017.01.008

[ref82] Wang Z. Lukowski S. L. Hart S. A. Lyons I. M. Thompson L. A. Kovas Y.. (2015). Is math anxiety always bad for math learning? The role of math motivation. Psychol. Sci. 26, 1863–1876. doi: 10.1177/0956797615602471, PMID: 26518438 PMC4679544

[ref83] Wang Z. Shakeshaft N. Schofield K. Malanchini M. (2018). Anxiety is not enough to drive me away: a latent profile analysis on math anxiety and math motivation. PLoS One 13:e0192072. doi: 10.1371/journal.pone.0192072, PMID: 29444137 PMC5812593

[ref84] Wanous J. P. Reichers A. E. Hudy M. J. (1997). Overall job satisfaction: how good are single-item measures? J. Appl. Psychol. 82, 247–252. doi: 10.1037/0021-9010.82.2.247, PMID: 9109282

[ref85] West C. P. Dyrbye L. N. Sloan J. A. Shanafelt T. D. (2009). Single item measures of emotional exhaustion and depersonalization are useful for assessing burnout in medical professionals. J. Gen. Intern. Med. 24, 1318–1321. doi: 10.1007/s11606-009-1129-z, PMID: 19802645 PMC2787943

[ref86] White H. (1980). A heteroskedasticity-consistent covariance matrix estimator and a direct test for heteroskedasticity. Econometrica 48, 817–838. doi: 10.2307/1912934

[ref87] Wigfield A. Cambria J. (2010). Students’ achievement values, goal orientations, and interest: definitions, development, and relations to achievement outcomes. Dev. Rev. 30, 1–35. doi: 10.1016/j.dr.2009.12.001

[ref88] Wigfield A. Eccles J. S. (2000). Expectancy-value theory of achievement motivation. Contemp. Educ. Psychol. 25, 68–81. doi: 10.1006/ceps.1999.1015 10620382

[ref89] Xie K. Vongkulluksn V. W. Lu L. Cheng S.-L. (2020). A person-centered approach to examining high-school students’ motivation, engagement and academic performance. Contemp. Educ. Psychol. 62:101877. doi: 10.1016/j.cedpsych.2020.101877

[ref1002] Yeager D. S. (2015). The National Study of Learning Mindsets, [United States], 2015-2016. Inter-university Consortium for Political and Social Research [distributor].

[ref90] Yeager D. S. Hanselman P. Walton G. M. Murray J. S. Crosnoe R. Muller C.. (2019). A national experiment reveals where a growth mindset improves achievement. Nature 573, 364–369. doi: 10.1038/s41586-019-1466-y, PMID: 31391586 PMC6786290

[ref91] Yerkes R. M. Dodson J. D. (1908). The relation of strength of stimulus to rapidity of habit-formation. J. Comp. Neurol. Psychol. 18, 459–482. doi: 10.1002/cne.920180503

[ref92] Zeidner M. (1998). Test Anxiety: The State of the Art Springer Science & Business Media.

